# Role of age in the association of chronic conditions and multimorbidity with incident ADL disability: multicohort analyses of 108,810 adults

**DOI:** 10.1007/s11357-025-01942-w

**Published:** 2025-11-14

**Authors:** Benjamin Landré, Aurore Fayosse, Louis Jacob, Mikaela Bloomberg, Ian Danilevicz, Frank van der Heide, Séverine Sabia, Archana Singh-Manoux

**Affiliations:** 1 Inserm U1153, Epidemiology of Ageing and Neurodegenerative Diseases, CRESS, Université Paris Cité, 10 Avenue de Verdun, Paris, 75010 France; 2 Department of Physical Medicine and Rehabilitation, Lariboisière-Fernand Widal Hospital, AP-HP, Université Paris Cité, Paris, France; 3 Research and Development Unit, Parc Sanitari Sant Joan de Déu, CIBERSAM, ISCIII, Dr. Antoni Pujadas, 42, Sant Boi de Llobregat, Barcelona, Spain; 4 Department of Behavioural Science and Health, University College London, London, UK; 5 UCL Brain Sciences, University College London, London, UK

**Keywords:** Multimorbidity, Disability, Ageing, Public health, Epidemiology

## Abstract

Chronic conditions and multimorbidity are increasingly prevalent in ageing populations, and their impact on disability remains poorly understood. This study investigated the association of chronic conditions and multimorbidity at ages 50, 60, 70, and 80 years with the subsequent onset of disability. Data from 108,810 participants (54% women, mean age 61.2 ± 9.8 at baseline) from the Health and Retirement Study, the English Longitudinal Study of Ageing, and the Survey of Health, Ageing, and Retirement in Europe were used. The number of chronic conditions was determined from a list of eight conditions. Cox regression was used to examine the incidence of disability in activities of daily living (ADL). ADL disability per 1000 person-years was 14.2 (13.7, 14.7) at age 50, 22.4 (21.9, 22.9) at age 60, 34.3 (33.4, 35.1) at age 70, and 67.6 (65.6, 69.6) at age 80. Having one chronic condition at age 50 was associated with a hazard ratio (HR) for ADL disability of 1.50 (1.42, 1.58) and 1.22 (1.13, 1.42) at age 80. For individuals with three or more chronic conditions, the HRs were 3.05 (2.79, 3.33) at age 50 and 1.74 (1.61, 1.87) at age 80. Analysis of population attributable fraction suggests that between 5.0 and 29.4% of incident disability, depending on the intervention scenarios and age at onset of chronic diseases, could be reduced by acting on chronic diseases. Chronic conditions and multimorbidity demonstrated a strong association with the onset of ADL disability, highlighting the need for early intervention to mitigate future disability risks.

**Supplementary Information:**

The online version contains supplementary material available at https://doi.org/10.1007/s11357-025-01942-w.

## Introduction

The twentieth century witnessed a striking demographic change such that over 21% of the world's population was 60 years or older by the year 2020 [1]. The ageing of populations worldwide presents tremendous societal challenges, particularly with the age-related increase in functional limitations, commonly measured using activities of daily living (ADL) [1]. The prevalence of ADL disability increases with age [1], highlighting the need to identify drivers of disability, particularly in early old age, where implementation of effective strategies to promote healthy ageing is still feasible.

The World Health Organization (WHO) framework for disability [2, 3] focuses on “intrinsic capacity”, defined as “the sum of all the individual’s physical and mental capacities” [4] for prevention of disability at older ages. The components or the best tools to measure intrinsic capacity remain to be established, not allowing strategies for enhancing or maintaining intrinsic capacity to be elaborated. An alternate approach proposes that chronic conditions play an important role in ageing outcomes such as disability [5]. Mortality from many chronic diseases has fallen sharply in recent decades [6], and their prevalence and that of multimorbidity [7, 8] have increased rapidly, starting in midlife [9]. Chronic diseases entail significant biological changes linked to ageing [10–12] that may also determine disability outcomes.

Incorporation of chronic diseases and multimorbidity in the disability framework is likely to yield a better understanding of health and functioning at older ages. In addition, health care provision could be structured to better manage the first chronic condition, with attention to the prevention of multimorbidity. Several studies provide evidence of a link between chronic diseases, multimorbidity, and incident disability in various contexts [13–18]. Whether this association exists across the adult age range and how it changes with the accumulation of chronic conditions remains unclear. These aspects of the association between multimorbidity and disability are important for effective prevention. Accordingly, our objective was to examine the association of the number of chronic conditions (one, two, three, or more) with incident ADL disability at ages 50, 60, 70, and 80 years using multicohort data from several developed countries with similar access to health care. For the populational impact on ADL disability, we model scenarios of the reduction in prevalence of chronic conditions and multimorbidity at different ages.

## Methods

### Data sources

Data were obtained from the Health and Retirement Study (HRS) [19], English Longitudinal Study of Ageing (ELSA) [20], and Survey of Health, Ageing, and Retirement in Europe (SHARE) [21] in populations from the USA, England, 26 European countries, and Israel, respectively. Details of survey designs can be found elsewhere [19–21].

Data from waves 2 to 15 from HRS (1994 to 2020), waves 1 to 9 from ELSA (2002 to 2018), and waves 1, 2, and 4 to 8 from SHARE (2004 to 2019) were obtained. The study baseline was defined at the first participation date in the waves retained in our analyses.

Participants included in the analyses were those without ADL disability at study baseline and with data from at least one follow-up wave. They were grouped into five regions for the analyses: Eastern Europe (Bulgaria, Croatia, Czech Republic, Estonia, Hungary, Latvia, Lithuania, Poland, Slovakia, Slovenia), Western Europe (Austria, Belgium, England, France, Germany, Luxembourg, Netherlands, Switzerland), Northern Europe (Denmark, Finland, Sweden), Southern Europe (Cyprus, Greece, Israel, Italy, Malta, Portugal, Spain), and North America (USA).

#### ADL disability

ADL disability was examined using six items of the Katz ADL scale (dressing, walking across a room, bathing/showering, eating, getting in/out of bed, and using the toilet). Participants were considered to have ADL disability if they reported difficulty in performing one or more of these activities for longer than 3 months.

#### Chronic conditions and multimorbidity

Chronic conditions consisted of self-reported medical diagnoses of eight common conditions that were available in all cohort studies: heart disease, hypertension, stroke, rheumatologic diseases, depression, lung diseases, cancer, and diabetes. Depression was also assessed using standardized questionnaires: EURO-D scores (SHARE) and CES-D (ELSA, HRS), in which missing data were imputed within the cohort using the CopyMean approach [22]. Participants were considered to have probable depression if their total score was above 3 for EURO-D [23] and 2 for CES-D [24]. For multimorbidity, participants were categorized as having two chronic conditions or three or more of the chronic conditions described above.

#### Covariates

Covariates included age, sex (male or female), marital status (married/cohabiting vs. others), education (categorized as primary [less than upper secondary or some high school], upper secondary [upper secondary or high school diploma and vocational training], or tertiary [university degree and above]), and household total wealth, also categorized in three groups based on tertiles, defined separately in each country.

#### Statistical analysis

Data from all individual studies were pooled for the analyses. The characteristics of participants at baseline were described overall and by disability status at the end of the follow-up.

We first examined the association of the number of chronic conditions (none, 1, 2, 3, or more) with incident ADL disability in the total sample using incidence rates, their differences, and Cox regression analysis. Chronic condition status was considered as a time-varying measure. The Cox regression model was adjusted for age, sex, marital status, education, household wealth, and region (East, West, South, North Europe, and the USA). To verify the robustness of the results, we repeated these analyses using a meta-analysis approach, based on a random effects model using the DerSimonian-Laird estimator method.

We then extracted the number of chronic conditions and covariates at ages 50, 60, 70, and 80 years (± 5 years at each age) for each participant and examined associations with incident ADL disability in separate Cox regression models. The follow-up began at the wave when data on chronic conditions were extracted at ages 50, 60, 70, and 80, respectively, and participants were censored at the report of ADL disability or end of follow-up, whichever came first.

In subsequent analyses, we derived population attributable fraction (PAF) [25] and potential impact fraction (PIF) [26] for the impact on ADL disability of three hypothetical scenarios consisting of reductions in prevalence of any chronic condition, two or more chronic conditions, and three or more chronic conditions. These analyses were undertaken in participants aged 50, 60, 70, and 80. PAF and PIF estimates in our analyses show the hypothetical reduction in incident ADL over a 4-year time frame for either complete removal (PAF) or reduction (PIF) of exposure specified in hypothetical scenarios. These estimates were calculated using a 4-year follow-up as this length of follow-up was available for all age groups (50, 60, 70, and 80 years), allowing comparison of estimates across the age groups. The PAF and PIF estimates were expressed as a percentage, with confidence intervals calculated using 250 bootstrap replications.

We used R software (version 4.3.0) for all the analyses. All estimates are reported with a 95% CI, and two-tailed *P* values were considered to be significant at the 0.05 level.

## Results

 The pooled sample included 108,810 participants, with 25,182 (23.1%) participants from HRS, 9,730 (8.9%) from ELSA, and 73,898 (67.9%) from SHARE. Table 1 shows baseline characteristics of participants overall and as a function of incident ADL disability (yes, n=30,894, 28.4%; no, n=77,916, 71.6%) at the end of the follow-up. The overall mean age at baseline was 61.2 (± 9.8) years, and 53.8% of participants were women. Overall, 40.1% of participants had no chronic condition at baseline, 17.1% had two, and 8.4% had three or more. The proportion with ADL disability at the end of the follow-up was highest in Western Europe (8917/35600, 25.1%) and lowest in Northern Europe (1535/8911, 17.2%), Table 1.

**Table 1:** Baseline characteristics of participants as a function of disability status at the end of the follow-up

	Total (N = 108,810)	No ADL disability (N = 77,916)	ADL disability (N=30,894)	*P* value
Age, mean (SD)	61.2 (9.8)	60.4 (9.4)	63.0 (10.3)	< 0.001
Women, *N* (%)	58,589 (53.8%)	40,666 (52.2%)	17,923 (58.0%)	
Education, *N* (%)				< 0.001
Primary	35,749 (32.9%)	24,466 (31.4%)	11,283 (36.5%)	
Upper secondary	47,820 (43.9%)	33,657 (43.2%)	14,163 (45.8%)	
Tertiary	25,241 (23.2%)	19,793 (25.4%)	5448 (17.6%)	
Household wealth, *N* (%)				< 0.001
Low	35,687 (32.8%)	24,197 (31.1%)	11,490 (37.2%)	
Medium	37,540 (34.5%)	26,471 (34.0%)	11,069 (35.8%)	
High	35,583 (32.7%)	27,248 (35.0%)	8335 (27.0%)	
Chronic diseases, *N* (%)				< 0.001
No disease	43,681 (40.1%)	34,659 (44.5%)	9022 (29.2%)	
One disease	37,306 (34.3%)	26,456 (34.0%)	10,850 (35.1%)	
Two diseases	18,653 (17.1%)	11,744 (15.1%)	6909 (22.4%)	
Three or more diseases	9170 (8.4%)	5057 (6.5%)	4113 (13.3%)	
ADL disability				
Eastern Europe, *N*(%)	20,925 (100%)	16,371 (78.2%)	4554 (21.8%)	
Bulgaria	657 (100%)	562 (85.5%)	95 (14.5%)	
Croatia	1588 (100%)	1285 (80.9%)	303 (19.1%)	
Czech R	4360 (100%)	3136 (71.9%)	1224 (28.1%)	
Estonia	4569 (100%)	3282 (71.8%)	1287 (28.2%)	
Hungary	1135 (100%)	942 (83.0%)	193 (17.0%)	
Latvia	545 (100%)	450 (82.6%)	95 (17.4%)	
Lithuania	870 (100%)	750 (86.2%)	120 (13.8%)	
Poland	2221 (100%)	1819 (81.9%)	402 (18.1%)	
Romania	833 (100%)	717 (86.1%)	116 (13.9%)	
Slovakia	806 (100%)	754 (93.5%)	52 (6.5%)	
Slovenia	3341 (100%)	2674 (80.0%)	667 (20.0%)	
Western Europe	35,600 (100%)	26,683 (75.0%)	8917 (25.0%)	
Austria	3826 (100%)	2875 (75.1%)	951 (24.9%)	
Belgium	5494 (100%)	4081 (74.3%)	1413 (25.7%)	
England	9730 (100%)	6730 (69.2%)	3000 (30.8%)	
France	4827 (100%)	3713 (76.9%)	1114 (23.1%)	
Germany	4347 (100%)	3313 (76.2%)	1034 (23.8%)	
Luxembourg	1179 (100%)	929 (78.8%)	250 (21.2%)	
Netherland	3205 (100%)	2670 (83.3%)	535 (16.7%)	
Switzerland	2992 (100%)	2372 (79.3%)	620 (20.7%)	
Northern Europe	8911 (100%)	7376 (82.8%)	1535 (17.2%)	
Denmark	3855 (100%)	3230 (83.8%)	625 (16.2%)	
Finland	901 (100%)	827 (91.8%)	74 (8.2%)	
Sweden	4155 (100%)	3319 (79.9%)	836 (20.1%)	
Southern Europe	18,192 (100%)	14,613 (80.3%)	3579 (19.7%)	
Cyprus	395 (100%)	325 (82.3%)	70 (17.7%)	
Greece	4201 (100%)	3510 (83.6%)	691 (16.4%)	
Israel	2198 (100%)	1789 (81.4%)	409 (18.6%)	
Italy	4742 (100%)	3755 (79.2%)	987 (20.8%)	
Malta	589 (100%)	568 (96.4%)	21 (3.6%)	
Portugal	959 (100%)	791 (82.5%)	168 (17.5%)	
Spain	5108 (100%)	3875 (75.9%)	1233 (24.1%)	
United States	25,182 (100%)	12,873 (51.1%)	12,309 (48.9%)	

*ADL* activities of daily living, *SD* standard deviation

The median follow-up overall was 6.0 years (interquartile range 3.0, 9.0), and the incidence rate of ADL disability was 43.4 per 1000 person-years (Table 2). Incidence rates of ADL disability in the total study population varied as a function of the number of chronic conditions, from 24.0 in participants without chronic conditions to 31.5, 37.2, and 47.8 per 1000 person-years in those with one, two, and three or more chronic conditions, respectively. Using “no chronic condition” as the reference, one (hazard ratio [HR] 1.48 [95% confidence interval 1.43, 1.52]), two (1.98 [1.91, 2.05]), or three or more chronic conditions (2.56 [2.47, 2.65]) were associated with a higher risk of incident ADL disability in adjusted analyses (Table 2).

**Table 2:** Association between the number of chronic conditions (0, 1, 2, 3, or more) and risk of incident ADL disability, in the total study population and using information at specific ages

	**Number of chronic diseases**	***N* cases of ADL disability**	**Total *N***	**Years of follow-up, median (IQR)**	**Incidence rate (95% CI) per 1000 person-years**	**Incidence rate differences (95% CI)**	**Hazard ratio (95% CI)**
All^a^	Total study sample	30,894	108,810	6.0 (3.0, 9.0)	43.4 (42.9, 43.9)	NA	NA
	No chronic condition	5486	43,681	4.0 (2.0, 7.0)	24.0 (23.3, 24.6)	Ref	Ref
	One chronic condition	9458	49,638	5.0 (2.0, 8.0)	31.5 (30.9, 32.2)	7.5 (6.6, 8.4)	1.48 (1.43, 1.52)
	Two chronic conditions	8454	33,131	6.0 (3.0, 9.0)	37.2 (36.4, 38.0)	13.3 (12.2, 14.3)	1.98 (1.91, 2.05)
	Three or more chronic conditions	7496	19,614	7.0 (4.0, 11.0)	47.8 (46.7, 48.9)	23.8 (22.3, 25.1)	2.56 (2.47, 2.65)
	**Analyses using information at key ages**						
50 ± 5 years^b^	All	3141	31,486	6.0 (3.0, 10.0)	14.2 (13.7, 14.7)	NA	NA
	No chronic condition	998	15,393	6.0 (4.0, 11.0)	8.4 (7.9, 8.9)	Ref	Ref
	One chronic condition	1129	10,197	6.0 (3.0, 10.0)	16.0 (15.1, 16.9)	7.6 (6.5, 8.7)	1.50 (1.42, 1.58)
	Two chronic conditions	655	3955	6.0 (2.5, 8.0)	26.9 (24.9, 29.1)	18.5 (16.4, 20.7)	2.18 (2.05, 2.32)
	Three or more chronic conditions	359	1395	4.0 (2.0, 7.0)	50.5 (45.4, 56.0)	42.1 (36.8, 47.3)	3.05 (2.79, 3.33)
60 ± 5 years^b^	All	6595	51,485	4.0 (2.0, 8.0)	22.4 (21.9, 22.9)	NA	NA
	No chronic condition	1492	18,247	4.0 (2.0, 8.0)	13.2 (12.6, 13.9)	Ref	Ref
	One chronic condition	2173	18,131	4.0 (2.0, 8.0)	20.6 (19.8, 21.5)	7.4 (6.3, 8.5)	1.37 (1.31, 1.43)
	Two chronic conditions	1728	9971	4.0 (2.0, 7.0)	33.0 (31.4, 34.6)	19.7 (18.1, 21.4)	1.80 (1.72, 1.89)
	Three or more chronic conditions	1202	5136	3.0 (2.0, 6.0)	50.6 (47.8, 53.5)	37.4 (34.4, 40.3)	2.39 (2.26, 2.53)
70 ± 5 years^b^	All	6306	37,846	3.0 (2.0, 6.0)	34.3 (33.4, 35.1)	NA	NA
	No chronic condition	887	8911	4.0 (2.0, 8.0)	18.1 (16.9, 19.3)	Ref	Ref
	One chronic condition	1931	12,820	4.0 (2.0, 7.0)	29.9 (28.5, 31.2)	11.8 (10.0, 13.6)	1.35 (1.29, 1.42)
	Two chronic conditions	1817	9384	3.0 (2.0, 6.0)	42.4 (40.5, 44.4)	24.3 (22.0, 26.6)	1.69 (1.60, 1.78)
	Three or more chronic conditions	1671	6731	3.0 (2.0, 5.0)	61.2 (58.3, 64.2)	43.2 (40.0, 46.3)	2.18 (2.06, 2.30)
80 ± 5 years^b^	All	4262	17,199	3.0 (2.0, 4.0)	67.6 (65.6, 69.6)	NA	NA
	No chronic condition	490	2635	3.0 (2.0, 6.0)	43.8 (40.0, 47.8)	Ref	Ref
	One chronic condition	1150	5258	3.0 (2.0, 5.0)	56.1 (52.9, 59.4)	12.2 (7.2, 17.3)	1.22 (1.13, 1.32)
	Two chronic conditions	1251	4768	3.0 (2.0, 4.0)	74.1 (70.1, 78.4)	30.4 (24.7, 36.0)	1.46 (1.35, 1.58)
	Three or more chronic conditions	1371	4538	2.0 (2.0, 4.0)	94.5 (89.6, 99.7)	50.7 (44.4, 57.1)	1.74 (1.61, 1.87)

*CI* confidence interval, *IQR* interquartile range (Q1–Q3), *NA* not applicable

^a^Cox regression in the total study sample using the number of chronic diseases entered as a time-varying measure, adjusted for age, sex, education, household wealth, and region

^b^Separate Cox regressions using the number of chronic diseases and covariates at specific ages and adjusted for age (within each age range), sex, education, household wealth, and region

**Fig. 1:**
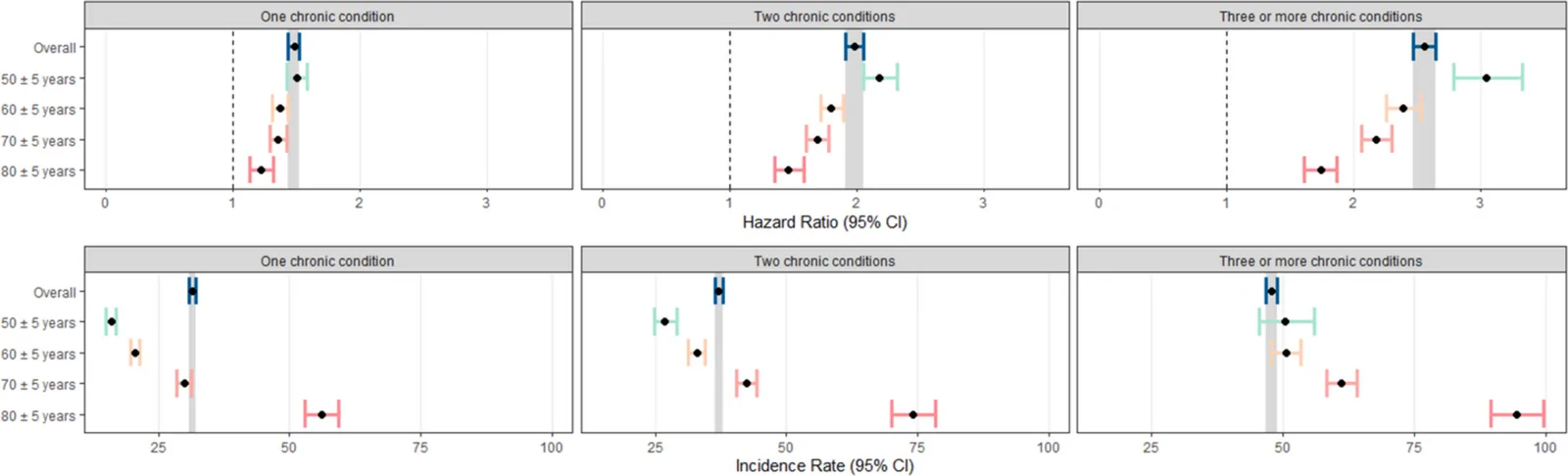
Association (hazard ratio (top panel) and incidence rate^a^ (lower panel)) of the number of chronic conditions (none, 1, 2, 3, or more) with incident ADL disability overall^b^ and as a function of age at baseline^c^. Abbreviations: CI, confidence interval. ^a^IR expressed per 1000 person-years, with 95% confidence intervals. ^b^Cox regression in the total study sample using the number of chronic conditions entered as a time-varying measure, adjusted for age, sex, education, household wealth, and region. ^c^Separate Cox regressions using the number of chronic conditions and covariates at ages 50, 60, 70, and 80 years, analyses adjusted for age (within each age range), sex, education, household wealth, and region. The grey area corresponds to the CI of the hazard ratio in the total study sample

Incident ADL disability rates were higher at older ages: 14.2, 22.4, 34.3, and 67.6 per 1000 person-years in those aged 50, 60, 70, and 80 years, respectively (Table 2). Incident ADL disability rates were also higher in those with a greater number of chronic conditions, irrespective of age. It is worth noting that participants with three or more chronic conditions at age 50 had a higher incident ADL disability rate (50.5) than participants without chronic conditions at age 80 (43.8). The absolute differences between participants with no chronic conditions and those with three or more chronic conditions were similar in all age groups: 42.1, 37.4, 43.2, and 50.7 in those 50, 60, 70, and 80 years, respectively. The hazard ratios for incident ADL disability were systematically higher in those with a greater number of chronic conditions, and the relative gradient from none to three or more diseases was stronger at younger ages (Table 2; Fig. 1, top panel). For example, participants with three or more chronic conditions at age 50 had a HR of 3.05 (2.79, 3.33) for incident ADL disability, whereas in the 80-year group, the HR in those with three or more chronic conditions was 1.74 (1.61, 1.87) (Table 2). For completeness, the lower panel in Fig. 1 shows incident rates per 1000 person-years in those with one, two, and three or more chronic conditions.

In additional analyses at the level of individual countries, the association of chronic conditions with incident ADL disability was similar to that in the main pooled analysis (Fig. S1, Table S1). The estimates from pooled and meta-analyses were similar in the total study population (Table S1). Using data at the level of the region (Table S1), we found the HRs to be highest in Northern Europe, although the incidence of disability over the follow-up was lowest in this group (Table 1).

The results of the impact on incident ADL disability of complete removal of chronic conditions (at least one, two, or three chronic conditions), expressed using the population attributable fraction (PAF), are shown in Table 3. The PAF estimates targeting those with at least one chronic condition were highest due to the prevalence of this condition being highest in all age groups. As expected, the PAF estimates for targeting multimorbidity (both at least two or at least three chronic conditions) were lower due to the lower prevalence of these conditions. For example, in participants who were 50 years old, the prevalence of at least one chronic condition was 49.4%, whereas that of at least three conditions in this group was only 4.4% (Table 3).

**Table 3:** The population attributable fraction (PAF) associated with targeting chronic conditions for incident ADL disability

	**Proportion in sample**	**Hazard ratio (95% CI)**^**d**^	**PAF (95% CI)**^**e**^
Targeting those with at least 1 chronic condition^a^ at			
50 ± 5 years	49.4% (15,547)	1.78 (1.70, 1.86)	27.1 (25.1, 29.1)
60 ± 5 years	64.6% (33,238)	1.64 (1.58, 1.71)	27.8 (26.0, 29.6)
70 ± 5 years	76.5% (28,935)	1.64 (1.57, 1.72)	29.4 (27.1, 31.8)
80 ± 5 years	84.7% (14,564)	1.46 (1.37, 1.57)	22.7 (18.5, 26.8)
Targeting those with at least 2 chronic conditions^b^ at			
50 ± 5 years	17.0% (5,350)	1.98 (1.88, 2.09)	13.9 (12.8, 15.0)
60 ± 5 years	29.3% (15,107)	1.70 (1.64, 1.76)	15.7 (14.6, 16.8)
70 ± 5 years	42.6% (16,115)	1.58 (1.52, 1.63)	17.3 (16.0, 18.6)
80 ± 5 years	54.1% (9,306)	1.41 (1.35, 1.48)	14.2 (12.2, 16.2)
Targeting those with at least 3 chronic conditions^c^ at			
50 ± 5 years	4.4% (1,395)	2.26 (2.08, 2.45)	5.0 (4.4, 5.6)
60 ± 5 years	10.0% (5,136)	1.83 (1.75, 1.92)	6.9 (6.5, 7.5)
70 ± 5 years	17.8% (6,731)	1.64 (1.57, 1.71)	8.5 (7.7, 9.4)
80 ± 5 years	26.4% (4,538)	1.42 (1.35, 1.47)	7.3 (6.1, 8.4)

*PAF* population attributable fraction, *CI* confidence interval

^a^Reference group for the hazard ratio was participants with no chronic condition

^b^Reference group for the hazard ratio was participants with none or one chronic condition

^c^Reference group for the hazard ratio was participants with none or up to two chronic conditions

^d^Hazard ratio for incident ADL disability in age-specific analysis (50 ± 5, 60 ± 5, 70 ± 5, and 80 ± 5 years) using separate Cox regressions with chronic condition status and covariates drawn at specific ages (± 5 years), analyses adjusted for age (within each age category), sex, education, household wealth, and region

^e^Estimated for follow-up at year 4, confidence intervals estimated using 250 bootstrap replications

**Fig. 2:**
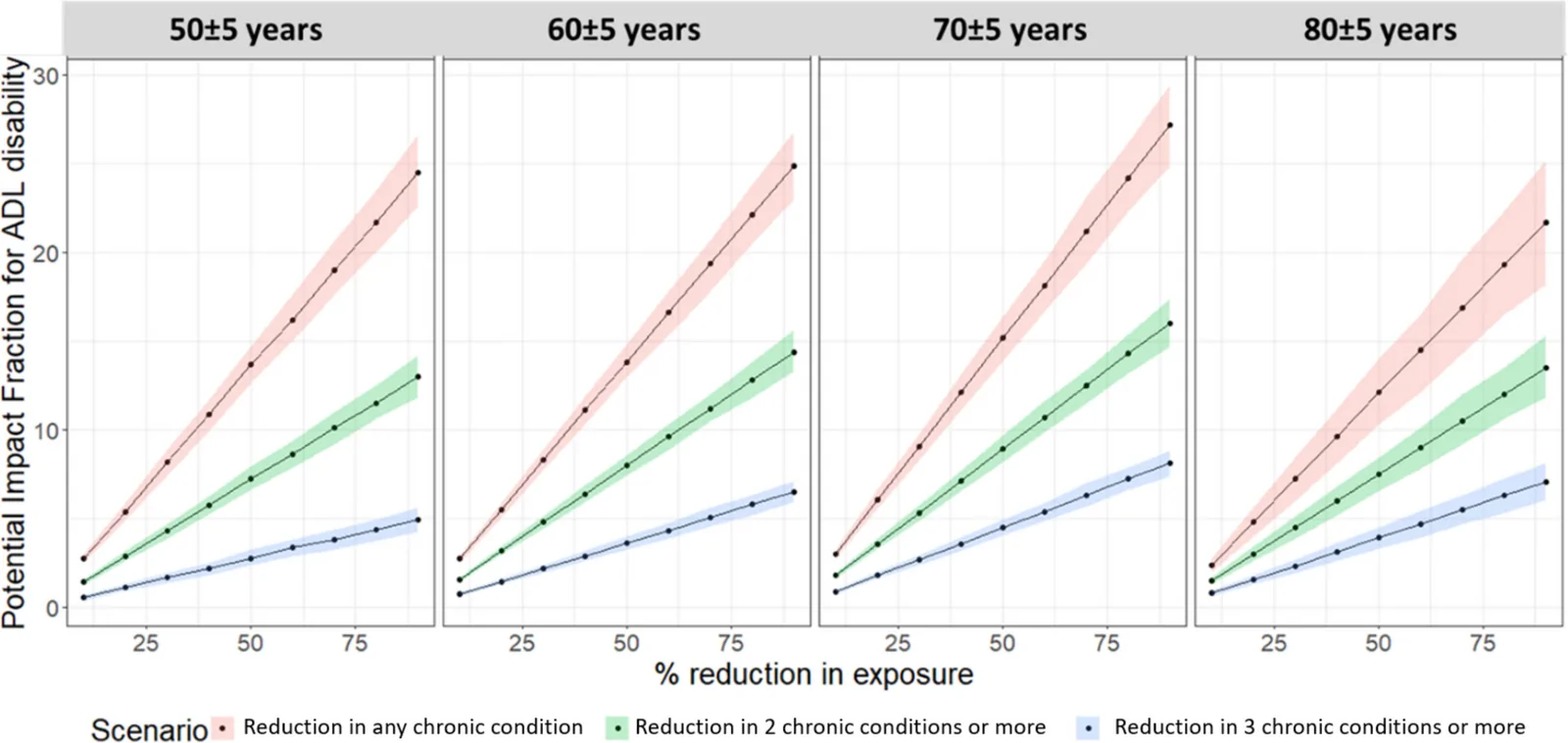
Impact of reduction in the prevalence of chronic conditions (any chronic condition, two chronic conditions or more, three chronic conditions or more) on ADL disability—estimations using potential impact fraction by age groups. Estimated for follow-up at year 4, based on Cox regression using chronic condition status and covariates at ages 50, 60, 70, and 80 years. Analyses adjusted for age (within each age group), sex, education, household wealth, and region; confidence intervals estimated using 250 bootstrap replications

As PAF estimates reflect the unrealistic scenario requiring complete removal of the exposure, we also used the potential impact fraction (PIF) that can be useful to model scenarios that reflect a reduction in the prevalence of the exposure (Fig. 2, Table S2). A 50% reduction in exposure would hypothetically lead to incident ADL disability being reduced by 13.7%, 13.8%, 15.2%, and 12.1% at ages 50, 60, 70, and 80 years, respectively, in those with any chronic condition; by 7.3%, 8.4%, 8.9%, and 7.5% at ages 50, 60, 70, and 80 years, respectively, in those with two or more chronic conditions; and 2.8%, 3.6%, 4.5%, and 3.9% at ages 50, 60, 70, and 80 years, respectively, in those with three or more chronic conditions.

## Discussion

Autonomy at older ages is a much desired individual and societal goal, the latter because of the health and social care costs of caring for people with disability in ageing populations. To better understand the role of chronic diseases in the onset of ADL disability, we undertook a study on 108,810 participants from prospective cohort studies from Europe and the USA. We report three key findings. One, chronic conditions and multimorbidity had a robust association with incident ADL disability, with associations being strongest in those with multiple chronic conditions. Two, using measures of ADL disability defined identically across the age range, we found the hazard ratios to be highest in those with a chronic condition and multimorbidity at age 50 and lowest in those aged 80. Three, within the prevention framework, our results suggest that complete removal or the more realistic scenarios of reduction in prevalence of multimorbidity would have a considerable impact on reductions in incident ADL disability across the adult lifespan.

Our results on the incidence of disability being higher at older ages and in those with chronic conditions are broadly consistent with a recent meta-analysis of 32 studies (total *N* 71,135) that examined the prevalence of disability in adults aged over 60 years and reported disability rates to increase with age and to be higher in those with multimorbidity [27]. A systematic review of 37 studies published in 2015 reported multimorbidity to be associated with functional decline over a period ranging from 1 to 6 years in adults over 18 years [28]. Our study adds to this literature by examining incidence rather than prevalence of disability and considering the role of age at onset of chronic diseases and multimorbidity in the longitudinal associations with disability. A striking finding in our study is the rapid increase in incidence of disability, often attributed to an old age outcome, in those with multiple chronic conditions, irrespective of age. The relative risk of ADL disability being highest in the youngest age group in our analyses highlights the need to prioritize interventions to improve functioning outcomes in middle-aged adults with chronic conditions. Given the rapid increase in multimorbidity, even by age 50, there is an urgent need to reconsider disability as a central health outcome on par with mortality, particularly for people with multiple chronic conditions.

An important focus of our study is multimorbidity due to an increase in its prevalence and younger age at onset [7]. Our results provide robust evidence of the risk of ADL disability in adults with multimorbidity, irrespective of the age of the person. Identification of the risk factors of ADL disability is important because changing disability status after its onset is challenging; a scoping review of nonpharmacological interventions on disability in community-dwelling older adults found them to have little or no effect [29]. It is within this framework that PAF and PIF estimates were used in our study to allow reflection on action on chronic conditions to reduce the risk of ADL disability for three distinct scenarios: targeting all chronic conditions, multimorbidity defined as two or more and three or more chronic diseases. Both PAF and PIF depend on the prevalence of the exposure (chronic disease/multimorbidity) and its association with the outcome (ADL disability). PAF and PIF assume a causal relationship between chronic diseases and ADL disability, a hypothesis that is not possible to prove in observational studies. Nevertheless, there is considerable research that suggests connections between chronic diseases and ageing outcomes [5, 30], and our findings highlight the existence of these associations across the adult age span.

Studies typically only estimate PAF, the estimated reduction in cases that would have resulted from the removal of the exposure. In our analyses, removal of all chronic conditions yielded a PAF between 22.7 (exposure at age 80) and 27.1% (exposure at age 50). The PIF estimates provide more realistic scenarios, based on the reduction rather than the elimination of an exposure. The PIF estimates better reflect real-life scenarios of public health interventions that target chronic disease. A 50% reduction in multimorbidity at age 50, defined as two or more chronic conditions, would result in a 7.3% reduction in ADL disability. Prevention of multimorbidity is feasible and promoted in “guidelines for people not for diseases” [31]. This approach offers possibilities of interventions at the level of the individual (i.e. patient coaching) and the care provider (i.e. review of all medications, patient-centred care) [32] and can be implemented at the onset of a first chronic condition to prevent multimorbidity [29, 33].

We chose limitations in ADLs [34] to define disability, as is common in research in this domain, particularly in international comparisons [1]. ADL disability is thought to offer a simple and clinically meaningful measure of disability. In our study, the same measure of ADL disability was used across the age groups and all cohort studies to allow conclusions to be drawn on the impact of chronic diseases and multimorbidity on incident ADL disability at ages 50, 60, 70, and 80 years. Whether an objective measure of disability would yield similar results to those in our study remains unclear due to limited data that would allow similar analyses to those reported here.

The mechanisms linking chronic diseases and multimorbidity to incident ADL disability are likely to involve biological, behavioural, and psychosocial pathways [5, 35, 36]. At older ages, when there is a marked increase in clustering of chronic conditions in individuals, it is possible that side effects of treatment for chronic conditions [35, 37] also play a role. Emerging research on the biology of ageing in terms of hallmarks of ageing (genomic instability, telomere attrition, epigenetic alterations, and cellular senescence) [10–12], identification of proteomic biomarkers of age-related disorders [38], and inflammatory processes [39, 40] is likely to provide insight into common pathways linking chronic diseases and functioning. The interdisciplinary research on resilience, focusing on adaptability across the life course to life stressors [36], may also be useful to better understand the link between chronic disease and disability.

A major strength of our study lies in the substantial sample size, allowing the investigation of the role of age in the association between multimorbidity and ADL disability. This was possible because these three large cohort studies on ageing are fairly similar in design. In addition, we were able to use the same definition of chronic conditions, multimorbidity, and ADL disability across the age range in the analyses. The findings of our study need to be considered in light of several limitations. One, all chronic conditions were self-reported, and the possibility of misclassification cannot be ruled out, although people are generally aware of their chronic diseases. Two, only a restricted set of eight common chronic conditions could be examined, and it is possible that other chronic diseases have a somewhat different pattern of associations. The choice of these eight chronic conditions in our study was guided by the availability of data across multiple cohort studies to allow sufficient statistical power in the analyses on the role of age and avoiding an exclusive focus on conditions that are rare in midlife (e.g. sensory impairments). These decisions imply that our results do not reflect the prevalence of chronic diseases or multimorbidity between 50 and 80 years, and other studies [7] with a focus on prevalence are better suited for this purpose. Three multimorbidity patterns may differ across age and regions [41, 42]. There is some evidence from previous studies to suggest that, for disability, the specific multimorbidity pattern does not affect associations with disability [43], but further research is needed to examine whether the mechanisms underlying these associations differ. Fourth, participants in the oldest age category were small in number and had a shorter follow-up. However, the follow-up within each age category did not differ as a function of the number of chronic conditions, implying that the association between chronic conditions and ADL disability is unlikely to be biased by the length of follow-up.

In conclusion, the findings of our study support the hypothesis that chronic conditions and multimorbidity are major drivers of ADL disability. Multimorbidity is an important target for the prevention of disability, particularly as the association between multimorbidity and ADL disability is not confined to old age. Prevention of chronic diseases is a public health priority and endorsed by several international guidelines [44–47], and our findings suggest that targeting middle-aged adults might yield benefits for outcomes such as disability.

## Supplementary Information

Below is the link to the electronic supplementary material.

ESM 1 (PDF 339 KB)

## Data Availability

English Longitudinal Study of Ageing data are freely available to researchers through the UK Data Service. Survey of Health, Ageing and Retirement in Europe (SHARE) data are accessible after registration with the SHARE project. Health and Retirement Study data are available upon registration with the University of Michigan at https://hrsdata.isr.umich.edu/data-products/public-survey-data.
