# Supplementary material for: Role of age in the association of chronic conditions and multimorbidity with incident ADL disability: multicohort analyses of 108,810 adults
Source: GeroScience. 2025 Nov 14;48(4):6005–16. doi: 10.1007/s11357-025-01942-w (PMC13575073; doi:10.1007/s11357-025-01942-w)
Supplement: Supplementary file 1 [file 11357_2025_1942_MOESM1_ESM.pdf]

SUPPLEMENTARY DATA

Table S1. Association between the number of chronic conditions (0, 1, 2, 3 or more) and incident ADL disability, in the total study population and in individual cohort studies.[a]

	1 chronic condition HR (95% CI)	2 chronic conditions HR (95% CI)	3 or more chronic conditions HR (95% CI)
**All**			
**Pooled analysis[a]**	**1.48 (1.43, 1.52)**	**1.98 (1.91, 2.05)**	**2.56 (2.47, 2.65)**
**Meta-analysis[b]**	**1.47 (1.35, 1.59)**	**2.03 (1.88, 2.24)**	**2.66 (2.44, 2.87)**
**Eastern Europe[c]**	1.37 (1.26, 1.50)	1.88 (1.71, 2.06)	2.36 (2.15, 2.59)
Bulgaria	1.51 (0.83, 2.76)	3.01 (1.67, 5.42)	3.12 (1.52, 6.43)
Croatia	1.17 (0.84, 1.62)	1.83 (1.33, 2.51)	2.51 (1.80, 3.49)
Czech R	1.49 (1.24, 1.79)	2.35 (1.95, 2.82)	2.92 (2.41, 3.54)
Estonia	1.40 (1.17, 1.68)	2.01 (1.68, 2.41)	2.69 (2.25, 3.22)
Hungary	1.02 (0.66, 1.56)	1.02 (0.65, 1.61)	1.81 (1.22, 2.69)
Latvia	1.52 (0.94, 2.47)	1.57 (0.92, 2.67)	1.98 (1.11, 3.56)
Lithuania	1.58 (1.00, 2.51)	2.50 (1.49, 4.22)	2.67 (1.51, 4.72)
Poland	1.37 (1.01, 1.86)	1.71 (1.27, 2.32)	2.02 (1.49, 2.73)
Romania	1.66 (1.12, 2.46)	2.25 (1.40, 3.61)	1.78 (0.69, 4.61)
Slovakia	1.63 (0.81, 3.30)	4.24 (2.01, 8.94)	6.32 (2.49, 16.04)
Slovenia	1.39 (1.12, 1.73)	1.70 (1.35, 2.13)	2.39 (1.87, 3.05)
**Western Europe[c]**	1.48 (1.39, 1.57)	2.04 (1.92, 2.17)	2.60 (2.44, 2.78)
Austria	1.46 (1.22, 1.74)	1.67 (1.38, 2.02)	2.68 (2.20, 3.26)
Belgium	1.36 (1.17, 1.59)	1.91 (1.64, 2.24)	2.69 (2.28, 3.17)
England	1.45 (1.32, 1.60)	1.97 (1.77, 2.18)	2.47 (2.21, 2.77)
France	1.66 (1.36, 2.03)	2.56 (2.09, 3.14)	2.87 (2.33, 3.54)
Germany	1.54 (1.28, 1.85)	2.34 (1.95, 2.83)	2.78 (2.27, 3.39)
Luxembourg	1.35 (0.94, 1.94)	1.60 (1.09, 2.34)	2.11 (1.44, 3.10)
Netherland	1.38 (1.11, 1.71)	1.76 (1.39, 2.24)	2.07 (1.59, 2.71)
Switzerland	1.57 (1.27, 1.94)	2.09 (1.66, 2.64)	2.51 (1.94, 3.25)
**Northern Europe[c]**	1.50 (1.29, 1.73)	2.14 (1.84, 2.50)	2.93 (1.49, 3.44)
Denmark	1.47 (1.16, 1.86)	2.10 (1.64, 2.69)	3.28 (2.53, 4.25)
Finland	0.71 (0.37, 1.38)	1.63 (0.85, 3.12)	2.84 (1.51, 5.37)
Sweden	1.64 (1.35, 1.99)	2.28 (1.86, 2.80)	2.75 (2.21, 3.41)
**Southern Europe[c]**	1.48 (1.34, 1.63)	1.76 (1.59, 1.95)	2.34 (2.10, 2.61)
Cyprus	1.33 (0.74, 2.40)	1.48 (0.72, 3.07)	3.80 (2.07, 6.97)
Greece	1.30 (1.05, 1.62)	1.66 (1.32, 2.09)	2.02 (1.58, 2.58)
Israel	1.28 (0.97, 1.70)	1.55 (1.15, 2.09)	2.60 (1.96, 3.46)
Italy	1.78 (1.45, 2.18)	1.80 (1.45, 2.24)	2.54 (2.05, 3.15)
Malta	3.96 (0.84, 18.73)	5.61 (1.17, 27.24)	6.52 (1.04, 40.83)
Portugal	2.10 (1.39, 3.19)	2.55 (1.66, 3.93)	2.79 (1.71, 4.56)
Spain	1.30 (1.10, 1.54)	1.66 (1.40, 1.97)	2.10 (1.75, 2.52)
**United States[c]**	1.48 (1.41, 1.56)	1.97 (1.87, 2.07)	2.52 (2.39, 2.66)

[a]Pooled analysis - Cox regression in total study sample using the number of chronic diseases (0, 1, 2, 3 or more) entered as a time varying measure, analyses adjusted for age, sex, education, household wealth, and region.
[b]Meta-analysis – random effects models using the DerSimonian-Laird estimator method of country-specific results.
[c]Country-specific analysis - Cox regression using the number of chronic diseases entered as a time varying measure, analyses adjusted for age, sex, education, and household wealth.

Table S2. Impact of reduction (from 10% to 90%) in chronic conditions on ADL disability – estimations using Potential Impact Fraction (PIF).

	SCENARIOS OF REDUCTION OF EXPOSURE								
	**10%**	**20%**	**30%**	**40%**	**50%**	**60%**	**70%**	**80%**	**90%**
**PIF (95% CI) for reduction in exposure in group with 1 or more chronic conditions**									
50±5 years	2.7 (2.5, 3.0)	5.4 (5.0, 5.9)	8.2 (7.5, 8.9)	10.9 (10.0, 11.7)	13.7 (12.6, 14.7)	16.2 (15.0, 17.6)	19.0 (17.6, 20.6)	21.7 (20.0, 23.5)	24.5 (22.6, 26.6)
60±5 years	2.8 (2.5, 3.0)	5.5 (5.1, 6.0)	8.3 (7.7, 8.9)	11.1 (10.3, 11.9)	13.8 (13.0, 14.8)	16.6 (15.3, 17.8)	19.4 (17.8, 20.7)	22.1 (20.5, 23.8)	24.9 (23.0, 26.8)
70±5 years	3.0 (2.7, 3.3)	6.1 (5.6, 6.6)	9.1 (8.3, 9.8)	12.1 (11.1, 13.1)	15.2 (13.9, 16.4)	18.1 (16.7, 19.5)	21.2 (19.4, 23.1)	24.2 (22.3, 26.2)	27.2 (24.9, 29.4)
80±5 years	2.4 (2.0, 2.8)	4.8 (4.0, 5.6)	7.3 (6.0, 8.4)	9.7 (8.1, 11.2)	12.1 (10.3, 14.0)	14.5 (12.1, 16.5)	16.9 (14.3, 19.6)	19.3 (16.5, 22.3)	21.7 (18.2, 25.2)
**PIF (95% CI) for reduction in exposure in group with 2 or more chronic conditions**									
50±5 years	1.4 (1.2, 1.6)	2.9 (2.6, 3.2)	4.3 (3.9, 4.7)	5.8 (5.3, 6.3)	7.3 (6.6, 8.0)	8.6 (7.9, 9.4)	10.1 (9.8, 11.0)	11.5 (10.6, 12.5)	13.0 (11.8, 14.2)
60±5 years	1.6 (1.5, 1.7)	3.2 (3.0, 3.4)	4.8 (4.4, 5.2)	6.4 (6.0, 6.9)	8.0 (7.4, 8.5)	9.6 (8.9, 10.3)	11.2 (10.5, 12.0)	12.8 (11.8, 13.8)	14.4 (13.3, 15.6)
70±5 years	1.8 (1.6, 2.0)	3.6 (3.3, 3.9)	5.3 (4.9, 5.8)	7.1 (6.5, 7.7)	8.9 (8.2, 9.7)	10.7 (9.9, 11.6)	12.5 (11.5, 13.4)	14.3 (13.2, 15.4)	16.0 (14.7, 17.4)
80±5 years	1.5 (1.3, 1.7)	3.0 (2.6, 3.4)	4.5 (3.9, 5.0)	6.0 (5.2, 6.8)	7.5 (6.6, 8.5)	9.0 (7.8, 10.1)	10.5 (9.2, 12.0)	12.0 (10.6, 13.5)	13.5 (11.8, 15.2)
**PIF (95% CI) for reduction in exposure in group with 3 or more chronic conditions**									
50±5 years	0.6 (0.4, 0.7)	1.2 (1.0, 1.4)	1.7 (1.4, 2.0)	2.2 (1.8, 2.5)	2.8 (2.4, 3.2)	3.4 (2.9, 3.8)	3.8 (3.3, 4.4)	4.4 (3.8, 5.0)	4.9 (4.3, 5.6)
60±5 years	0.7 (0.6, 08)	1.5 (1.4, 1.7)	2.2 (1.9, 2.4)	2.9 (2.6, 3.2)	3.6 (3.3, 4.0)	4.3 (4.0, 4.8)	5.1 (4.6, 5.6)	5.8 (5.2, 6.3)	6.5 (5.9, 7.2)
70±5 years	0.9 (0.8, 1.0)	1.7 (1.5, 1.9)	2.7 (2.4, 3.0)	3.6 (3.2, 4.0)	4.5 (4.1, 5.0)	5.4 (4.9, 5.9)	6.3 (5.7, 7.0)	7.2 (6.6, 7.9)	8.1 (7.4, 8.8)
80±5 years	0.8 (0.6, 0.9)	1.5 (1.3, 1.8)	2.4 (2.0, 2.7)	3.1 (2.6, 3.6)	3.9 (3.3, 4.5)	4.7 (4.0, 5.4)	5.5 (4.7, 6.3)	6.3 (5.3, 7.2)	7.1 (6.1, 8.1)

Abbreviation: PIF: Potential Impact Fraction; CI: Confidence Interval.
Estimated for follow-up at year 4, based on Cox regression using chronic disease status and covariates at specific ages, analyses adjusted for sex, education, household wealth, and region; confidence intervals estimated using 250 bootstrap replications.

Figure S1: Association of chronic condition (1, 2, and 3 or more) with incident ADL disability overall (pooled and meta-analysis approach) and in each country.

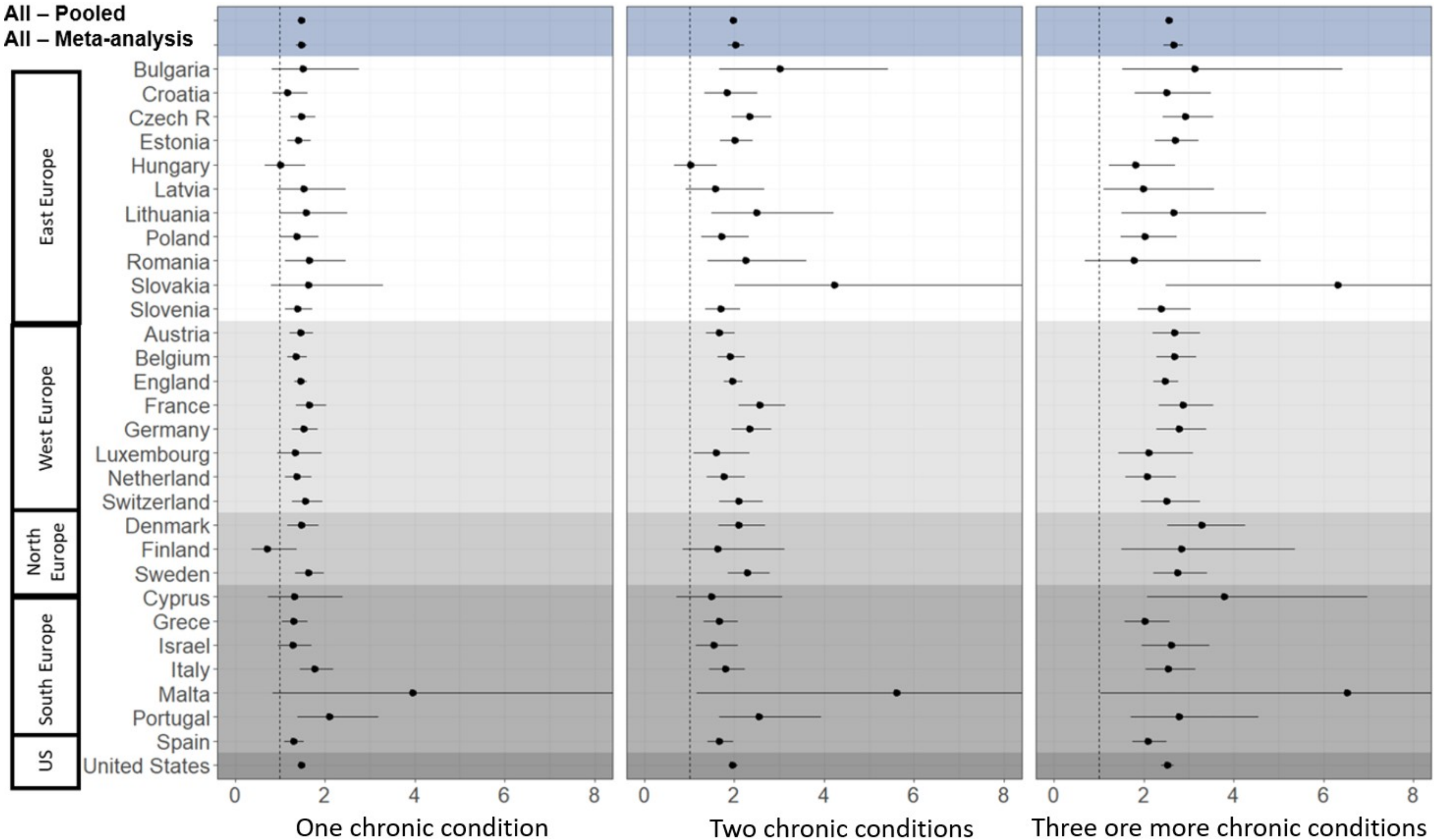

Pooled analysis - Cox regression in total study sample using the number of chronic conditions (0, 1, 2, 3 or more) entered as a time varying measure, analyses adjusted for age, sex, education, household wealth, and region.

Meta-analysis – random effects models using the DerSimonian-Laird estimator method of country-specific results.

Country-specific analysis - Cox regression using the number of chronic conditions entered as a time varying measure, analyses adjusted for age, sex, education, and household wealth
